# Condensation of delta‐1‐piperideine‐6‐carboxylate with ortho‐aminobenzaldehyde allows its simple, fast, and inexpensive quantification in the urine of patients with antiquitin deficiency

**DOI:** 10.1002/jimd.12214

**Published:** 2020-01-29

**Authors:** Thomas Boehm, Holger Hubmann, Karin Petroczi, Déborah Mathis, Kristaps Klavins, Guenter Fauler, Barbara Plecko, Eduard Struys, Bernd Jilma

**Affiliations:** ^1^ Department of Clinical Pharmacology Medical University of Vienna Vienna Austria; ^2^ Department of Pediatrics and Adolescent Medicine, Division of General Pediatrics Medical University of Graz Graz Austria; ^3^ Department of Clinical Chemistry and Biochemistry University Children's Hospital Zurich Zurich Switzerland; ^4^ CeMM Research Centre for Molecular Medicine of the Austrian Academy of Sciences Vienna Austria; ^5^ Clinical Institute of Medical and Chemical Laboratory Diagnostics Medical University of Graz Graz Austria; ^6^ Department of Clinical Chemistry Amsterdam University Medical Centers, location VUmc Amsterdam The Netherlands

**Keywords:** antiquitin deficiency, delta‐1‐piperideine‐6‐carboxylate, ortho‐aminobenzaldehyde, pyridoxal‐5‐phosphate, pyridoxine‐dependent epilepsy, α‐aminoadipic semialdehyde

## Abstract

Antiquitin (ATQ) deficiency leads to tissue, plasma, and urinary accumulation of alpha‐aminoadipic semialdehyde (AASA) and its Schiff base delta‐1‐piperideine‐6‐carboxylate (P6C). Although genetic testing of *ALDH7A1* is the most definitive diagnostic method, quantifications of pathognomonic metabolites are important for the diagnosis and evaluation of therapeutic and dietary interventions. Current metabolite quantification methods use laborious, technically highly complex, and expensive liquid chromatography‐tandem mass spectro‐metry, which is available only in selected laboratories worldwide. Incubation of ortho‐aminobenzaldehyde (oABA) with P6C leads to the formation of a triple aromatic ring structure with characteristic absorption and fluorescence properties. The mean concentration of P6C in nine urine samples from seven ATQ‐deficient patients under standard treatment protocols was statistically highly significantly different (*P* < .001) compared to the mean of 74 healthy controls aged between 2 months and 57 years. Using this limited data set the specificity and sensitivity is 100% for all tested age groups using a P6C cut‐off of 2.11 μmol/mmol creatinine, which represents the 99% prediction interval of the P6C concentrations in 17 control urine samples from children below 6 years of age. Plasma P6C concentrations were only elevated in one ATQ subject, possibly because P6C is trapped by pyridoxal‐5‐phosphate (PLP) blocking fusing with oABA. Nevertheless, both urine and plasma samples were amenable to the quantification of exogenous P6C with high response rates. The P6C quantification method using fusion of oABA with P6C is fast, simple, and inexpensive and might be readily implemented into routine clinical diagnostic laboratories for the early diagnosis of neonatal pyridoxine‐dependent epilepsy.

## 1 INTRODUCTION

Deficiency of antiquitin (ATQ; EC 1.2.1.31) is the most frequent cause of pyridoxine‐/vitamin B_6_‐dependent epilepsy. ATQ is encoded by *ALDH7A1* and mutations compromising dehydrogenase activity lead to an accumulation of alpha‐aminoadipic semialdehyde (AASA), which is in reversible equilibrium with the autocyclized Schiff base delta‐1‐piperideine‐6‐carboxylate (P6C^1^). Both AASA and P6C are elevated in the urine and plasma of ATQ‐deficient patients.^2^, ^3^, ^4^, ^5^ Elevated levels are decreased during pyridoxine treatment, lysine restricted diet and arginine supplementation (van Karnebeek et al^6^, ^7^). A Knoevenagel reaction‐based fusion of P6C with pyridoxal‐5‐phosphate (PLP) and secondary depletion of PLP is likely involved in the clinical symptomatology of affected individuals.^1^

The state‐of‐the‐art quantification methods of AASA and P6C using plasma and urine are based on liquid chromatography‐tandem mass spectrometry (LC‐MS/MS) methods.^3^, ^4^, ^5^, ^8^ Quantification of AASA/P6C is not readily available in routine clinical laboratories, both because of the expensive equipment needed and the expertise required to run these highly technological instruments. Only a few laboratories worldwide are able to quantify AASA and P6C to support the diagnosis of ATQ deficiency.^8^, ^9^ Early diagnosis through the implementation of a simple and inexpensive P6C quantification method into routine clinical chemistry laboratories might help to reduce diagnostic uncertainty and thus minimise severe neurodevelopmental consequences in ATQ‐deficient patients.

Fusion of ortho‐aminobenzaldehyde (oABA) with delta‐1‐pyrroline or delta‐1‐piperideine, the autocyclized oxidation products after deamination of putrescine and cadaverine, respectively, generates a triple aromatic ring structure.^10^, ^11^, ^12^ We hypothesized that oABA might also form a similar condensation product with P6C.

Here, we show that the absorption properties of the fusion product between oABA and P6C allow simple, fast, and inexpensive quantification of elevated P6C concentrations in the urine of ATQ‐deficient patients. The only necessary equipment is a spectrophotometer or a microplate reader with a sensitive absorption measurement module.

## 2 METHODS

### 2.1 Chemical synthesis of P6C

The synthesis of P6C is based on a method by Reference 13 and is described in Reference 1. The P6C concentration was adjusted to 1 mM using LC‐MS/MS‐based quantification^4^ and small aliquots were stored at −30°C.

### 2.2 Analysis of endogenous and exogenous (spiked) P6C in urine

Thawed urine samples were centrifuged at 10 000 rpm for 5 minutes and 90 μL of the supernatant diluted with 100 μL water and either 10 μL 10% ethanol (negative control or reference control for the oABA matrix) or 10 μL 20 mM oABA (A9628, Sigma‐Aldrich, Vienna, Austria) were added. For the quantification of exogenous P6C 10 μL of concentrated P6C at different concentrations and 90 μL water were used. All samples were analysed in duplicate. oABA was dissolved in 100% ethanol and stored for approximately 6 months at −30°C without an increase in polymerisation or a noticeable reduction of performance. The 200 μL reaction mixture was incubated in the dark for 2 hours at room temperature. Absorption was measured in a Synergy H1 Multi‐Mode Microplate reader (BioTek, Winooski, Vermont) reader and scans performed using 4 nm steps. The absorption values at 460 nm or the mean of the values at 456/460/464 nm obtained during absorptions scans from 300 to 600 nm were used for calculations. The resolution of the Synergy H1 for absorbance measurements is 0.0001 optical density units.

The first block of measurements including repeated measurements of all ATQ deficiency samples and 19 adult healthy controls was performed in April/May 2019. The mean (SD) slope of the four standard curves representing the apparent extinction coefficient was 4035 (668) M^−1^ cm^−1^ (Table S2). Additional controls including more adults, as well as children and adolescents of different ages, were measured in October 2019 using the same P6C standard and the same healthy volunteer urine samples frozen in single‐use aliquots. Nevertheless, the mean (SD) apparent extinction coefficient of three experiments decreased to 2517 (204) M^−1^ cm^−1^, implying degradation of the P6C standard. The difference between the extinction coefficients measured in May and October is statistically significant (*P* = .013). To be able to properly compare the April/May data with that from October, we adjusted the October data using a mean factor of 1.61 (see Table S2 for calculations). It might be possible to perform this assay without P6C standard, because the extinction coefficient under specified conditions is an intrinsic parameter. The relative difference between controls and ATQ deficient patients in a given experiment is independent of the extinction coefficient. Nevertheless, proper reactivity of oABA is tested with the P6C standard.

A shift of the entire absorption curve by a few nm was observed in less than 5% of wells using UV half‐area 96‐well microtiter plates. Two examples are shown in Figure S8 and the consequences are described in Tables S3 and S4. Under most circumstances, the results will not be relevantly influenced, but in combination with low creatinine concentrations the apparent P6C concentrations can falsely double or even triple. This may be due to a batch problem with our UV plates, but for automated analysis similar observations with other microtiter plates must be properly corrected.

### 2.3 ATQ‐deficient patients and healthy volunteers

A total of nine urine samples from seven ATQ patients collected at two hospitals (Graz, Austria and Zurich, Switzerland) were included. Relevant patient and sample characteristics are described in Table 1. In April/May, we tested eight “old” urine samples stored for 3 years at −30°C and 11 “fresh” urine samples which had been taken from healthy adult volunteers and stored for less than 4 weeks at −30°C. In October, we tested a further 11 “fresh” urine samples taken from healthy volunteers and stored for less than 4 weeks at −30°C. We combined the adult samples in the cohort >17 years of age (cohort >17 HV in Table 1), because there was no difference between the three sub‐cohorts. In October 2019, we measured the P6C concentrations from 27 children and adolescents aged between 6 and 17 (cohort 6‐17 HV in Table 1), and 17 children below 6 years of age (cohort <6 HV). Some characteristics of the three control sample cohorts are listed in Table 1.

**Table 1:** Description of antiquitin (ATQ)‐deficient and healthy volunteer urine samples

Code	Age (years) at collection	Collection date^a^	Crea (mM)	AASA	P6C	Treatment	Mean P6C^b^	SD P6C^b^
Z1_1	6	23 Apr 19	5.8		0.5^c^	P, LRD	3.6	0.53
Z1_2	5	2 Jul 18	1.1	4.4^d^		P, LRD	9.5	2.19
Z2_1	5	1 Feb 19	1.1	1.5		P; LRD	2.9	0.56
Z2_2	4	30 Jan 18	2.9	0.7		P, LRD	4.1	1.13
Z3	2	14 Sep 17	5	5.5		P; LRD	3.8	0.82
Z4	0.3	17 May 17	0.8	19.9		P	12.3	2.16
G193	21	24 Feb 19	11.1	26^e^		P	3.9	0.43
G196	29	24 Feb 19	1.7	43		P	7.3	1.48
G205	1.2	24 Feb 19	4.4	26		P; LRD	5.1	0.58
>17 H^f^	36 (11.2)	Apr 16/19/Oct 19	8.4 (5.8)				0.36	0.22
6‐17 HV^f^	12.5 (3.3)	Oct 19	10.4 (7.3)				0.33	0.18
<6 HV^f^	2.2 (1.5)	Oct 19	3.4 (2.7)				0.80	0.45

Abbreviations: AASA, alpha‐aminoadipic semialdehyde; Crea, creatinine; LRD, lysine‐restricted diet; P, pyridoxine;P6C, delta‐1‐piperideine‐6‐carboxylate.

a For all samples collected in 2019 assays were performed within 4 weeks of collection and storage at −30°C.

b This study; μmol P6C/mmol creatinine; SD of the mean of the means of duplicate determinations of 4 to 5 independent experiments for ATQ‐deficiency patients (40 measurements) or 1 to 2 independent experiments for healthy volunteers (118 measurements).

c Method adapted from Reference 4; normal values <0.4 μmol P6C/mmol creatinine.

d Method adapted from Reference 1; normal values <1 μmol AASA/mmol creatinine.

e Method Graz Liquid chromatography‐mass spectrometry (LC–MS) based with Fmoc derivatization and deuterated alpha‐aminoadipic acid as internal standard; Normal values <14 μmol AASA/mmol creatinine (99% confidence interval of values measured in >100 healthy subjects; unpublished).

f >17 HV = urine samples from healthy volunteers (HV) >17 years old (n = 30); 6‐17 HV = samples from healthy children and adolescents (HV) between 6 and 17 years old (n = 27); <6 HV = samples from healthy children (HV) <6 years old (n = 17). The numbers in brackets are SDs for age in years and creatinine. P6C and creatinine concentrations are statistically highly significantly different between the adults or the age 6‐17 cohort and the children <6 years cohort with all *P*‐values <.001.

### 2.4 Ethics

All procedures were in accordance with the ethical standards of the responsible committee on human experimentation (institutional and national) and with the Helsinki Declaration of 1975, as revised in 2013. The ethics committees in Graz and Zurich allowed the use of the material without a formal vote, because the urine samples of ATQ‐deficiency patients, healthy children and adolescents were completely and irreversibly anonymized and collected during routine visits. All patients (or their parents) and all healthy volunteers provided their informed consent before the collection of blood and urine samples. The study numbers for the collection of blood and urine samples from healthy adult volunteers in Vienna are EK:2030/2013 and EK:1810/2015.

### 2.5 Statistics

Calculations of the three 99% prediction intervals in Figure 2 were performed using the means of n = 74 (all controls) and n = 17 (cohort below 6 years of age) samples with n−1° of freedom and the quantile *t*‐distribution to account for the estimation of the SDs. The 99% prediction intervals were derived using the means +2.648 (n = 74) or 2.921 (n = 17; 99% quantile) *SD of the means*sqrt(1 + 1/n). All three healthy volunteer populations described in Table 1 are normally distributed using the Kolmogorov‐Smirnov test statistics at a significance level of 0.01 with D (*P*) values of 0.162 (.362), 0.151 (.487), and 0.108 (.96) for the >17 HV, 6‐17 HV, and < 6 HV cohorts, respectively.

## 3 RESULTS

### 3.1 Absorption and fluorescence properties of the fusion product CHHPQ (oABA/P6C)

Incubation of oABA with delta‐1‐pyrroline (autocyclized form of oxidised putrescine), delta‐1‐piperideine (autocyclized form of oxidised cadaverine) or P6C generates triple aromatic ring structures with absorption maxima at 430 (5‐ring or pyrroline‐based) or 460 nm (6‐ring or piperideine‐based), respectively. The IUPAC name of the condensate between oABA and P6C is 9‐carboxy‐5,5a,6,7,8,9‐hexahydropyrido[2,1‐b]quinazoline‐10‐ium or abbreviated CHHPQ (see Figure S2E). Some absorption and fluorescence properties of CHHPQ generated after incubating oABA with P6C are presented in Figure S1. The postulated molecular weight and some corresponding MS2 fragments were identified using ESI mass spectrometry (Figure S2). Nevertheless, the structures must be considered provisional and verified using NMR or other suitable methods.

A typical P6C standard curve using fluorescence detection of the oABA/P6C condensate and recovery of spiking P6C into plasma of healthy volunteers and ATQ patients are shown in Figures S3A, S3B, and S3D. Quantification of P6C in plasma samples seems possible considering response rates of 80%, but only one ATQ patient showed elevated P6C concentrations (Figure S3C). All ATQ patients in this study received appropriate treatment, and free P6C concentrations are probably too low to use this method for quantification. In addition, it seems that the Knoevenagel condensate between PLP and P6C does not allow fusion with oABA, and excess PLP interferes with oABA/P6C fusion and quantification (Figure S4‐S6).

### 3.2 Urine is amenable for the measurement of the oABA/P6C condensation product

*ALDH7A1* mRNA and protein are highly expressed in the kidneys of mice and humans.^14^, ^15^ We hypothesized that P6C might be directly excreted into the urine, thus, explaining the generally elevated urine P6C concentrations when compared to plasma (1, 5, 7; Table S1). If P6C is directly excreted into urine, it might escape efficient trapping by PLP. About 90% of circulating PLP is bound to albumin and not filtrated into the urine before intracellular conversion to 4‐pyridoxic acid, which is found at high concentrations in urine but does not react with delta‐1‐pyrroline‐5‐carboxylic acid and is therefore unlikely to react with P6C.^16^, ^17^, ^18^ Experiments with pyridoxamine and pyridoxine did not provide evidence for any interaction with delta‐1‐pyrroline‐5‐carboxylic acid^16^ and it is improbable that P6C does.

**Figure 1:**
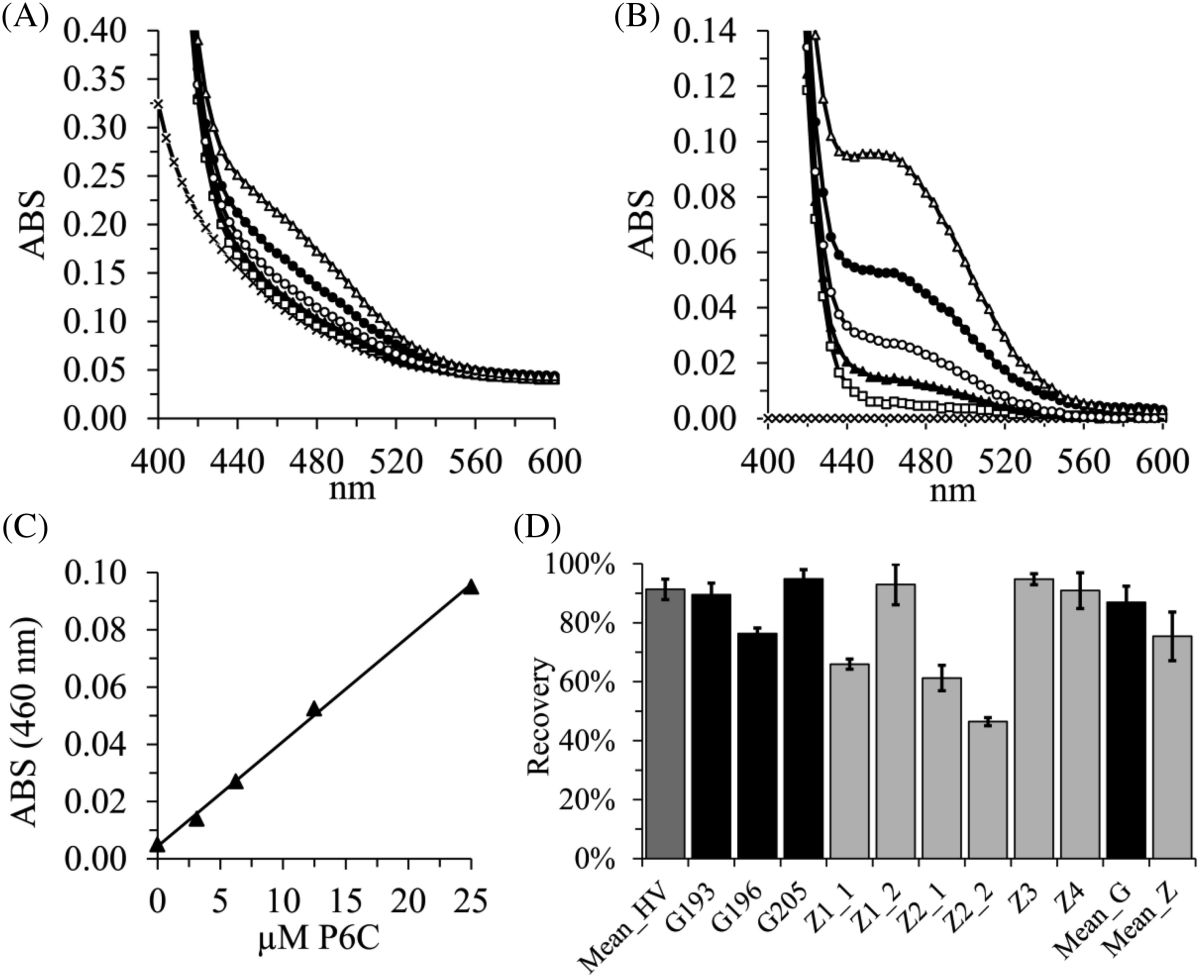
P6C concentrations can be precisely quantified in the urine samples of healthy volunteers and ATQ patients with high response rates of exogenously spiked P6C. A, Representative absorption scans of urine from a healthy volunteer (HV) after adding 0.5% ethanol (oABA matrix) or oABA and different concentrations of P6C; crosses (x) 0.5% ethanol; white squares (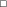) no P6C spiking; black triangles (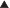) 3.1 μM; white circles (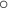) 6.3 μM; black circles (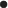) 12.5 μM and white triangles (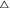) 25 μM P6C spiking; each symbol represents the mean of duplicates; B, These curves were generated by subtracting the 0.5% ethanol control from the curves shown in (A) representing specific oABA/P6C signal; C, The resulting standard curve from the data shown in (B) using absorption data at 460 nm; the regression line is y = 0.00366x + 0.0044; *R* = 0.998; D, Percent P6C response after exogenous P6C spiking presenting urinary data from 8 HVs (Mean_HV; two independent experiments in duplicate), nine individual samples from seven individual ATQ subjects and the means of the samples from Graz (Mean_G; three independent experiments in duplicate) and Zurich (Mean_Z; two independent experiments in duplicate); the means ± SE of the means (SEMs) are shown; ATQ, antiquitin; oABA, ortho‐aminobenzaldehyde; P6C, delta‐1‐piperideine‐6‐carboxylate

Figure 1A demonstrates the absorption spectra of a typical control urine sample from a healthy volunteer diluted 1 to 1 in water after the addition of 0.5% ethanol (oABA matrix), as well as different concentrations of P6C. In Figure 1B, the ethanol control absorption values have been subtracted to obtain the specific signal. The regression line of the representative standard curve using the absorption values at 460 nm is presented in Figure 1C. The endogenous P6C concentration in the urine of this healthy volunteer was 1.2 μM. The mean inter‐assay coefficient of variation (±SEM) of the duplicates of the standard curves in three independent experiments using six different P6C concentrations was 1.45% (0.35%). The mean inter‐assay coefficient of variation (±SEM) of the duplicates of the 27 to 40 samples, including ATQ patients and healthy volunteers in three independent experiments, was 1.79% (0.39%).

Response data of P6C after spiking 10 or 20 μM into the urine samples of healthy volunteers and ATQ patients are shown in Figure 1D. The mean response rate of ATQ samples is 80%, with one sample from Zurich repeatedly demonstrating response rates close to only 50%. This is also the patient with strong fluorescence quenching (see Supplement). These data demonstrate that a few μM P6C in urine can be readily quantified and that efficient condensation of 1 mM oABA with spiked P6C is possible in the urine of ATQ patients.

### 3.3 The oABA/P6C fusion product is highly elevated in urine samples of ATQ‐deficient patients

**Figure 2:**
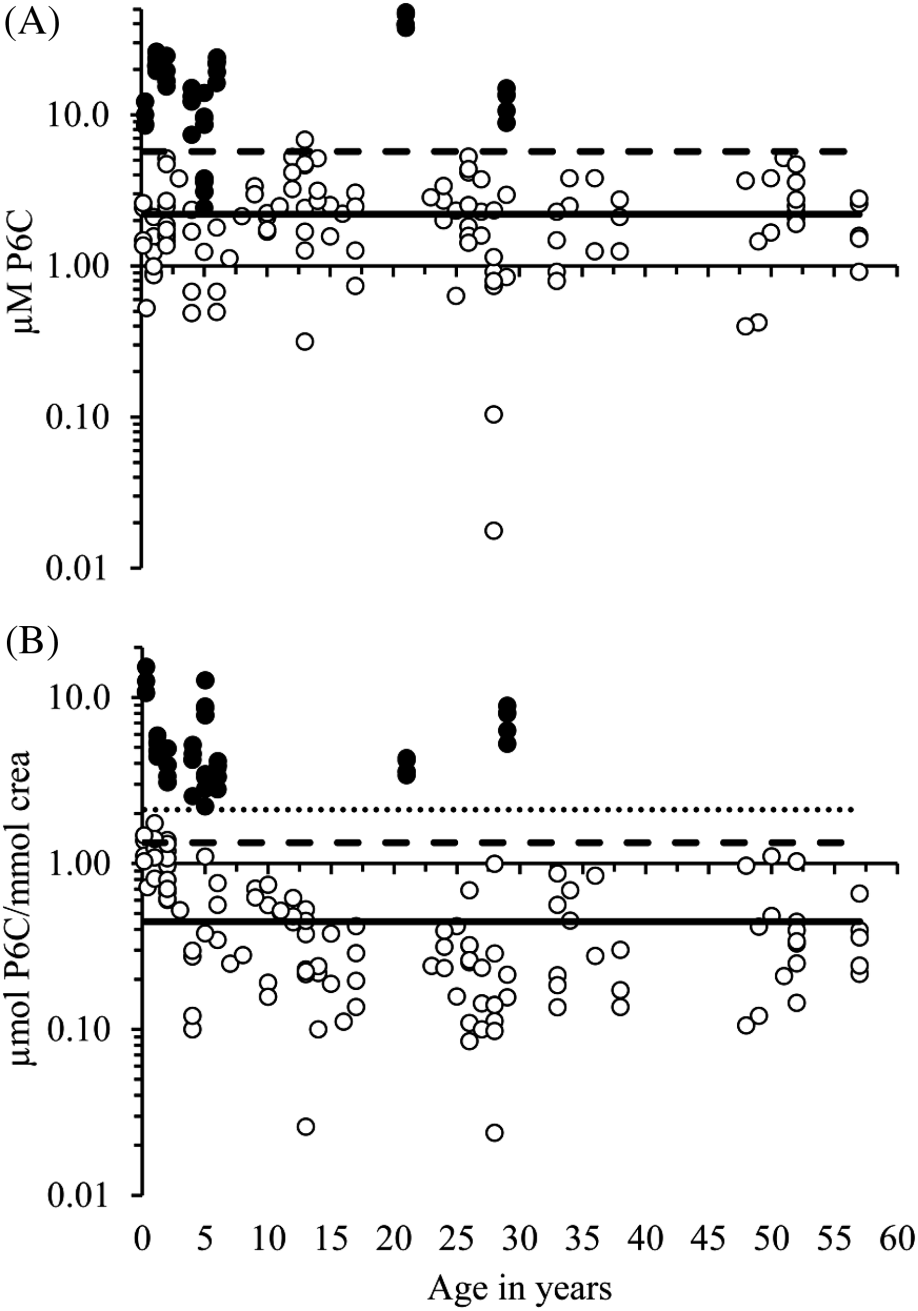
Urinary delta‐1‐piperideine‐6‐carboxylate (P6C) concentrations of nine samples from seven different antiquitin (ATQ) subjects are highly elevated compared to controls from 74 healthy volunteers of different ages. A, Six and three ATQ urine samples from Zurich and Graz, respectively, were measured 4‐ (Zurich) and 5‐ (Graz) times in duplicate in independent experiments; all 40 measurements are shown as black circles (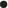); All 118 measurements from 74 urine samples from healthy volunteers (HV) of different ages are shown as white circles (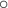); the solid and dashed lines represents the mean (2.2 μM) and the 99% prediction interval (PI; mean + 2.648*SD*sqrt(1 + 1/n); 5.71), respectively, from 74 HV urine samples; B, The data from (A) were converted to μmol P6C/mmol creatinine and the mean of all control samples (0.45) is shown as solid line. The dashed line at 1.33 represents the 99% PI (mean + 2.648*SD*sqrt[1 + 1/n]) of all urine control samples; the dotted line at 2.11 represents the 99% PI (mean + 2.921*SD*sqrt[1 + 1/n]) of only the control cohort samples from children below 6 years of age (n = 17)

The mean (SD) urinary endogenous P6C concentration in nine samples from 7 ATQ patients is 17.1 (11.7) μM or 5.9 (3.2) μmol/mmol creatinine (Figure 2A, B; n = 4‐5 independent experiments performed in duplicate and in total 40 measurements). The corresponding mean (SD) P6C concentrations in urine samples from the 74 healthy volunteers divided into three cohorts (Table 1) are 2.3 (1.13) μM and 0.36 (0.22) μmol/mmol creatinine for the adults, 2.6 (1.58) and 0.33 (0.18) for the children and adolescents aged between 6 and 17, and 1.9 (1.15) and 0.8 (0.45) for children below 6 years (Figure 2A and B). The P6C concentrations of the adult control cohort >17 HV and the cohort 6 to 17 HV are statistically highly significantly different (*P*‐values <.001) compared to the P6C concentrations in children below 6 years (cohort <6 HV). The P6C μM concentrations between the three control cohorts are not different. One contributing factor for the difference in the μmol P6C/mmol creatinine concentrations between the cohorts seems to be the creatinine concentration, which is statistically highly significantly different (*P*‐values <.001) comparing the >17 HV and the 6 to 17 HV with the <6 HV cohort. The correlation coefficient between age and creatinine using all children and adolescents (n = 44) is 0.63 with a *P*‐value of <.0001 (Figure S9).

The *P*‐values comparing the mean μM P6C and the mean μmol P6C/mmol creatinine concentrations between all healthy volunteers (n = 74) and ATQ patients (n = 9) are .0053 and .00096, respectively. The sensitivity and specificity using a P6C cut‐off of 2.11 μmol/mmol creatinine are 100% (Figure 2B). This value corresponds to the 99% prediction interval using only the 17 control urine samples from children below 6 years of age. The 99% prediction interval using all 74 control samples is 1.33 μmol/mmol creatinine (Figure 2B).

**Figure 3:**
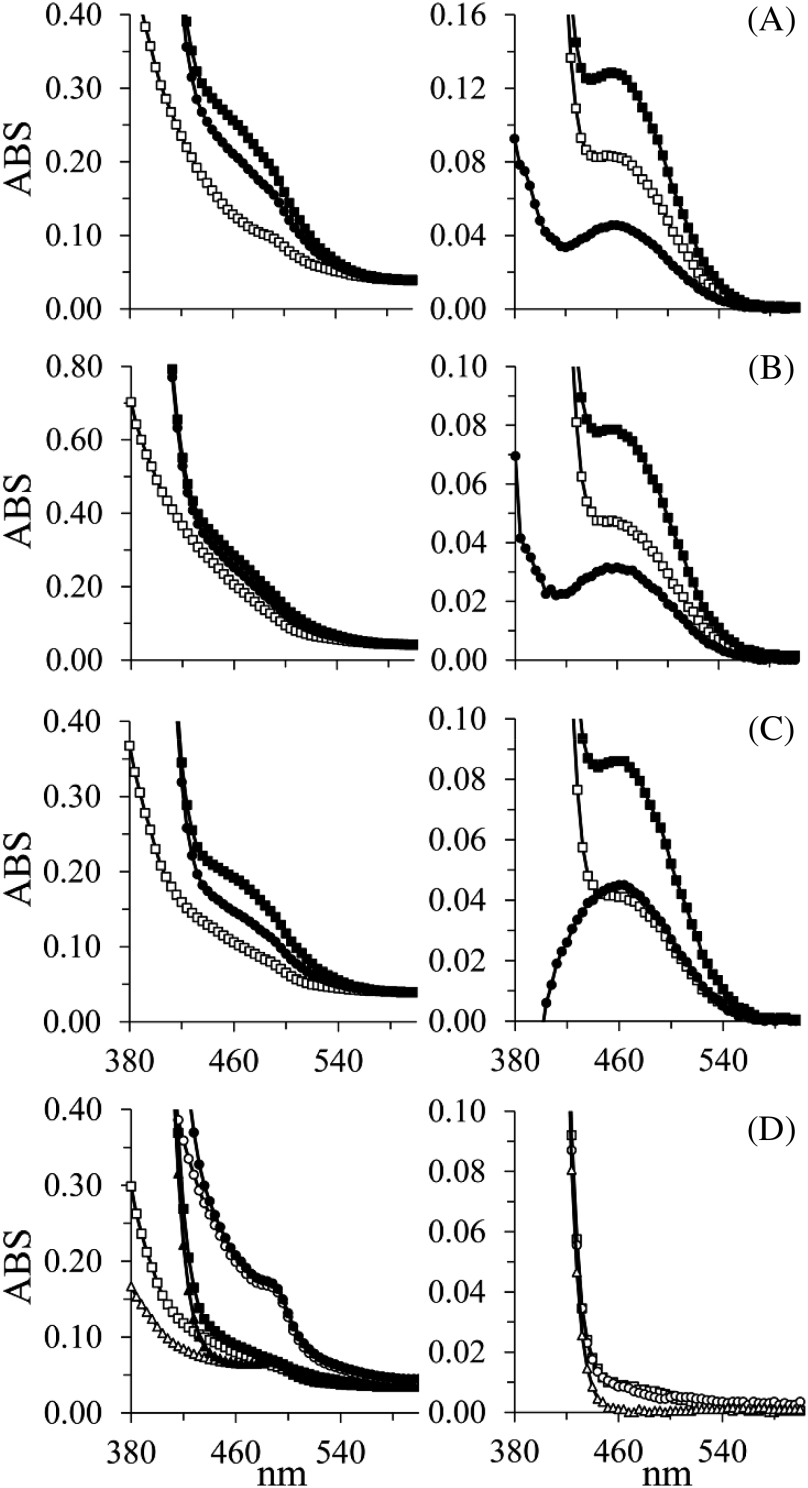
Examples of representative absorptions scans used for the quantification of endogenous and exogenous P6C in urine samples of ATQ and control subjects. Each symbol represents the mean of duplicates; G193 (A); Z1_1 (B) and Z3 (C) are data from 3 ATQ subjects and 3 healthy volunteers are shown in (D). In A‐C on the left side white squares (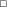) represent urine samples incubated with 0.5% ethanol; black circles (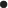) with oABA and black squares (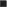) with oABA, and 10 μM P6C; in (D) white and black symbols represent incubation with ethanol and oABA, respectively; in A‐C on the right side white squares (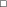) represent the specific endogenous P6C signal after subtracting the 0.5% ethanol from the oABA data; black circles (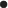) represent the specific signal after subtracting the oABA from the spiked P6C representing exogenous P6C and black squares (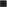) represent the signal after subtracting the ethanol from the exogenous P6C data representing both endogenous and exogenous P6C; in (D) the symbols represent endogenous P6C of three controls. ATQ, antiquitin; oABA, ortho‐aminobenzaldehyde; P6C, delta‐1‐piperideine‐6‐carboxylate

Representative individual absorption scan profiles of three ATQ patients and three control urine samples are shown in Figure 3. The oABA/P6C absorption profiles of endogenous and exogenous P6C generated after spiking synthesised P6C into ATQ patient urine samples are similar to control urine samples and the standard curves with exogenous P6C spiking.

Regression analysis of the P6C concentrations measured in Vienna with the AASA/P6C concentrations measured in Zurich is significant (*P* = .033) with a correlation coefficient of 85%. The correlation coefficient between the AASA concentrations measured in Graz and our method is 94%, but because we can only compare three samples the *P*‐value is not significant (*P* = .23). The Bland‐Altman plot method was not used because of limited sample sizes.

Some data and considerations about measuring the oABA/P6C condensate in urine using fluorescence detection are summarised in the Supplement and Figure S7. Experiments to quantify the condensate of P6C with oABA using fluorescence showed strong and variable quenching, resulting in a nonlinear response and erratic results in healthy volunteers and patients, thus, making a test using this characteristic without separation inadequate for diagnostic testing.

## 4 DISCUSSION

In 2016, about 134.6 million babies were born worldwide. Coughlin et al^19^ recently published data stating an estimated ATQ deficiency birth incidence of 1 in 64 352 live births, meaning that it is likely that in 2016, 2100 newborns developed pyridoxine dependent epilepsy. We do not know how many receive a correct and timely diagnosis. Any deferral in starting treatment with pyridoxine might cause more irreversible developmental delay.

Stockler et al^9^ suggested that a technically more simpler diagnostic method would facilitate diagnosis and newborn screening. Current LC‐MS/MS‐based methods are available in only a few laboratories worldwide.^8^, ^9^ Here, we report on a very simple and inexpensive method to measure P6C in the urine samples of ATQ‐deficient patients, which can be readily implemented in clinical chemistry laboratories worldwide, because only a simple and inexpensive chemical (ortho‐aminobenzaldehyde), urine, and a spectrophotometer or microplate reader with an absorption measurement module are necessary. The measured P6C concentrations in urine are comparable to the published LC‐MS/MS methods (Table S1). All patients tested in this study were treated with pyridoxine and some additionally with a lysine‐restricted diet, but the difference between determined control and patient urine P6C concentrations was statistically highly significant. Nevertheless, before this method can be used in clinical laboratories more data from healthy children below 3 months of age are necessary.

Within the limited data set of this study the specificity and sensitivity for all age groups is 100% using a P6C cut‐off of 2.11 μmol/mmol creatinine, which is equal to the 99% prediction interval for children below 6 years. The oABA‐based method for trapping and quantifying P6C before the start of therapeutic interventions might be even easier considering that P6C and AASA concentrations are significantly higher in untreated individuals.^5^, ^6^, ^8^ Recently, a novel biomarker for ATQ‐deficient patients was published, and indeed this offered better stability compared to P6C and AASA. However, the quantification method for this is still based on LC‐MS/MS.^20^

We only tested frozen urine samples and can only hypothesize that fresh urine samples from ATQ‐deficient patients and healthy controls might recapitulate the data. Yuzyuk et al^7^ reported a mean loss of 18% AASA/P6C signal using LC‐MS/MS after storage of urine for 120 hours at 4°C. Similar data were described using one ATQ‐deficiency urine sample.^8^ Therefore, if shipment of urine at 4°C is necessary but lasts less than a few days, the oABA condensation method might work. Nevertheless, this must be rigorously tested, because the background might increase. Alternatively, immediate addition of oABA might stabilise CHHPQ. Acidification of urine to pH 3 to 5, the optimal condition for oABA‐based condensation of delta‐1‐pyrroline or delta‐1‐piperideine, should be also tested.

It is critical that the P6C standard is stored at sufficiently low temperatures (below −70°C) and freshly prepared at appropriate intervals. A suitable commercial source of high‐quality P6C would be highly advantageous for clinical laboratories with limited chemistry expertise.

It is tempting to speculate that the quantification of P6C in plasma using oABA condensation might also be possible prior to starting treatment, and therefore our method might be suitable for newborn screening using urine and plasma. After the start of pyridoxine treatment, the concentration of free P6C and free AASA in plasma is significantly lower and therefore our method is not suitable for plasma P6C quantification. The Knoevenagel‐type condensation of PLP and P6C efficiently blocks absorption and fluorescence in vitro and possibly also in plasma. Farrant wrote that delta‐1‐pyrroline‐5‐carboxylic acid also condenses with aromatic and aliphatic aldehydes and ketones in vitro and P6C might do the same in vitro as well as in plasma. Therefore, we were surprised by some relatively high concentrations of P6C and AASA measured in plasma samples considering that AASA is a reactive aldehyde interacting with amino groups of proteins, and P6C might also interact with aldehydes and ketones. Nevertheless, P6C might already be efficiently trapped by PLP inside cells, where the PLP concentration is possibly much higher compared to plasma, even in most ATQ‐deficient patients. Our data suggest that mainly the PLP‐complexed form of P6C, or other P6C complexes, appear to be present in plasma. Median plasma PLP concentrations in healthy volunteers vs ATQ‐deficient patients are 72 and 779 nM, respectively,^21^ and in most ATQ individuals PLP per se will not interfere in the assay. The white blood cell intracellular concentration of PLP in healthy individuals is in the 10 to 50 μM range and is possibly 5 to 10‐fold increased in ATQ patients under high dose PLP treatment.^22^ Therefore, Knoevenagel‐type condensation reactions are more likely to happen inside cells. Nevertheless, it is possible that PLP interferes with oABA‐P6C condensation in plasma, although we do not consider this likely, because in most ATQ patients, PLP concentrations are below 1 μM. Is it possible that LC‐MS/MS‐based methods dissociate the Knoevenagel condensate or other complexes of aldehyde or ketones with P6C/AASA and thereby measure higher AASA/P6C concentrations?

Ortho‐aminobenzaldehyde‐based quantification in urine and plasma might be significantly improved using liquid chromatography before absorption and fluorescence measurements, but then some of this assay's simplicity would be lost. It might also be possible to increase absorption and fluorescence using oABA derivatives, which are still undergoing efficient fusion with P6C but show higher extinction coefficients and quantum yields.^23^

The elevated urinary AASA concentrations measured in molybdenum cofactor and sulphate oxidase deficiency might also be readily measurable using the oABA‐based quantification.^8^, ^24^

Flynn et al^25^ measured plasma and urine concentrations of delta‐1‐pyrroline‐5‐carboxylate (P5C) in Type II hyperprolinaemia patients, a rare disorder which presents with seizures, particularly during febrile illnesses.^16^, ^26^ Normal plasma values of P5C are in the 1 μM range using recombinant P5C reductase from *E. coli* for quantification.^27^ The mean (SD) P5C plasma concentration in five Type II hyperprolinaemia patients was 22 (5.5) μM. The baseline P5C concentration will also be measured using the oABA‐based method and therefore contributes to the oABA signal generated in healthy volunteers, but oABA/P6C‐based condensation might also detect Type II hyperprolinaemia in children with seizures of unknown cause. The extinction coefficient of the oABA/P5C condensation product is approximately 2400 and is similar to P6C.^28^, ^29^ Interestingly, Flynn et al.^25^ used an assay for quantification of P5C in urine based on oABA condensation, and reported that using urine the mean optical density value for control subjects was 0.01325 (n = 20). The mean optical density values for nine Type II hyperprolinaemia patients were massively elevated and ranged from 0.053 to 1.677, the latter number implying a urinary P5C concentration of 700 μM. The assay conditions were not described and therefore we cannot compare the data to the methods used in this study. Applegarth et al^26^ published a case report of Type II hyperprolinaemia with similar high urinary absorption data using oABA. Both the Flynn et al^25^ and Applegarth et al^26^ data strongly support the oABA‐based quantification method of P6C using urine from ATQ‐deficient patients.

## 5 CONCLUSION

Identification of ATQ deficiency and other rare diseases with elevated concentrations of P6C or P5C might be accomplished simply by adding oABA and matrix control to urine samples followed by sensitive absorption measurements. This method can easily be implemented in most clinical chemistry laboratories worldwide and might help to identify these potentially devastating diseases as early as possible. It is tempting to speculate that the clinical efficacy of new therapeutic interventions, such as the inhibition of upstream enzymes to reduce AASA/P6C concentrations, or ultimately even enzyme replacement therapies, might be evaluated in clinical trials with this simple, robust and inexpensive quantification of P6C and P5C using a chemical condensation reaction described more than 80 years ago.

## CONFLICT OF INTEREST

The authors declare no potential conflict of interest.

## AUTHORS CONTRIBUTIONS

TB designed the experiments, analysed the data and wrote the manuscript. KP performed most experiments and analysed the data; DM provided ATQ samples and quantified AASA and P6C in the Zurich samples; KK performed mass spectrometry experiments to identify CHHPQ and analysed the data; HH, GF, and BP provided ATQ and healthy children and adolescents samples and quantified AASA in the urine samples from Graz; ES synthesised P6C and quantified it; BJ provided critical comments during manuscript writing. All authors provided critical input during manuscript writing and were involved in the finalisation of the manuscript.

## Supporting information


**FIGURE S1** Absorption and fluorescence characterisation of the fusion product of P6C with oABA. **(A)** Absorption spectrum of 200 μM PC6 with and without preincubation with 1 mM oABA in 20 mM Hepes pH 7.0 for 2 hours at room temperature; **(B)** Excitation scan between 360 and 580 nm and fixed emission at 620 nm; **(C)** Excitation at 460 nm and emission scan between 500 and 700 nm; **(D)** Specific emission signal generated by subtracting the P6C/0.5% ethanol (oABA matrix) from the P6C/oABA RFUs; black squares (■) P6C incubated with oABA; white squares (□) P6C with ethanol (oABA matrix); black triangles (▲) H_2_O with oABA; white triangles (Δ) H_2_O with 0.5% ethanol; oABA, ortho‐aminobenzaldehyde; P6C, Delta‐1‐piperideine‐6‐carboxylate; RFU, Relative Fluorescence Units; ABS, Absorption
**Figure S2** Identification of the predicted P6C/oABA fusion product using ESI mass spectrometry. **(A)** P6C (m/z 128.1 Da) incubated with 0.5% ethanol (oABA matrix) in Hepes buffer; **(B)** P6C region enlarged; **(C)** P6C incubated with oABA with the expected new signal of the oABA/P6C condensate at 231.22 Da; the second new peak at 253.19 probably represents a sodium adduct (+22 Da); The main peaks at 239.16 and 260.87 Da are caused by Hepes and its sodium adduct; **(D)** MS/MS of the 231.22 Da peak with expected fragments of 106.1, 144.1 and 186.2 Da. **(E)** Relevant chemical structures including some MS2 fragments; oABA, ortho‐aminobenzaldehyde; P6C, Delta‐1‐piperideine‐6‐carboxylate; cps, counts per second
**Figure S3** P6C concentrations are not elevated in plasma of ATQ patients, but response rates of spiked P6C are high. **(A)** Example of a representative plasma standard curve after spiking different concentrations of P6C into EDTA plasma of a healthy volunteer (HV); the mean of duplicates is shown after subtracting fluorescence from the control sample with 0.5% ethanol (oABA matrix); R = 0.99; **(B)** Endogenous P6C concentrations in 7 HVs (black bars) and response after 20 μM P6C spiking (grey bars); Mean (+/‐SEM) response was 80% (9%); **(C)** P6C plasma concentrations of 9 samples from 7 different ATQ subjects (Zurich samples have been measured only once and Z1_2 only in singlicate; the mean (+/‐SEM) of the duplicates are shown; Graz samples have been measured once in duplicate and the mean (+/‐SEM) are shown) and healthy controls (HV; n = 7; different HV samples compared to (**B**) were used; G_C; n = 5; Graz control plasma samples from five subjects <18 years); the means of duplicates (+/‐SEM) are shown; **(D)** Response of spiked P6C in 4 ATQ subjects normalised to 3 HVs in one experiment and 2 HVs samples in a second experiment; we did not have enough plasma from the Zurich samples to perform further experiments; the means of duplicates (+/‐SEM) are shown; oABA, ortho‐aminobenzaldehyde; P6C, Delta‐1‐piperideine‐6‐carboxylate; RFU, Relative Fluorescence Units
**Figure S4** P6C Knoevenagel condensation with PLP does not allow subsequent oABA fusion. **(A)** 40 μM P6C was incubated with a 2‐ and 6‐fold excess of PLP overnight at 37°C followed by incubation with oABA for 90 minutes. Absorption was measured at pH 7.2; **(B)** Specific signal of **(A)** after subtracting the relevant controls; **(C)** Absorption measurements at pH 0.7; the low pH reduced the PLP and oABA signal about 5‐fold with a minimal effect on the P6C signal; **(D)** Specific signal of **(C)** after subtracting the relevant controls; In **(A)** to **(D)** white squares (□) represent P6C incubated with 0.5% ethanol (oABA matrix); black squares (■) P6C with oABA; black triangles (▲) P6C with a 2‐fold molar excess of PLP followed by oABA; black circles (●) P6C with a 6‐fold molar excess of PLP followed by oABA; crosses (x) just water with 0.5% ethanol; white triangles (Δ) and white circles (○) 80 and 240 μM PLP followed by oABA; **(E)** P6C was first incubated with oABA for 90 minutes at room temperature and afterwards a 10‐fold excess of PLP was added for 24 hours at 37°C before absorption measurements at room temperature and pH 0.7; white triangles (Δ) represent oABA in Hepes buffer; white squares (□) oABA incubated with PLP; black triangles (▲) oABA with P6C and black squares (■) oABA with P6C followed by a 10‐fold excess of PLP; **(F)** Summary of percent inhibition using absorption and fluorescence measurements at pH 7.2 and pH 0.7 incubating P6C first with oABA followed by PLP (oA_P6C_P) or P6C first with PLP followed by oABA after 24 hours (PC6_P_oA); Percentage inhibition was calculated using absorption data at 460 nm and fluorescence data with the custom filter cube after subtracting the appropriate controls; oABA, ortho‐aminobenzaldehyde; P6C, Delta‐1‐piperideine‐6‐carboxylate; PLP, Pyridoxal 5‐Phosphate; RFU, Relative Fluorescence Units; SD, SD; ABS, Absorption
**Figure S5** P6C Knoevenagel condensation with PLP does not allow subsequent oABA fusion ‐ Repeat. **(A)** 20 μM P6C was incubated with a 10‐fold excess of PLP for 24 hours at 37°C; 1 mM oABA was added for 90 minutes and absorption measured at pH 0.7; **(B)** Magnification of the 460 nm region of **(A)**; **(C)** same as **(A)** but measured at pH 7.2; **(D)** Magnification of the 460 nm region; white triangles (Δ) represent oABA with Hepes buffer (20 mM; pH 7.0); white squares (□) P6C incubation with oABA; black triangles (▲) P6C with a 10‐fold excess of PLP for 24 hours followed by oABA; black squares (■) PLP with oABA; oABA, ortho‐aminobenzaldehyde; P6C, Delta‐1‐piperideine‐6‐carboxylate; PLP, Pyridoxal 5′‐phosphate; ABS, Absorption
**Figure S6** PLP seems to be able to form a Knoevenagel condensate with the P6C/oABA fusion product and inhibits absorption and fluorescence. **(A)** 20 μM P6C was first incubated with oABA for 90 minutes at room temperature and afterwards a 10‐fold excess of PLP was added for 24 hours at 37°C and absorption measured at pH 0.7; **(B)** Magnification of the 460 nm region of **(A)** (also shown in Figure S4E but repeated here for consistency); **(C)** same as **(A)** but measured at pH 7.2; **(D)** Magnification of the 460 nm region of **(C)**; white triangles (Δ) represent incubation of oABA with Hepes buffer (20 mM; pH 7.0); white squares (□) oABA with PLP; black triangles (▲) oABA with P6C and black squares (■) oABA with P6C followed by a 10‐fold excess of PLP; oABA, ortho‐aminobenzaldehyde; P6C, Delta‐1‐piperideine‐6‐carboxylate; PLP, Pyridoxal 5′‐phosphate; ABS, Absorption
**Figure S7** Fluorescence‐based measurements of the oABA/P6C condensate are not straightforward in urine samples from ATQ patients. **(A)** The fluorescence standard curve after spiking different concentrations of exogenous P6C into the urine of a healthy volunteer; the control sample without addition of P6C was subtracted; 4PL non‐linear regression analysis (R = 0.99) was used to calculate the μM P6C concentrations of the ATQ subjects shown in (**B**); (**B)** Regression analysis between absorption and fluorescence μM concentrations excluding G205 and Z2_2 ATQ subject samples. Both show a negative signal after subtracting the ethanol autofluorescence signal from the signal in the presence of oABA indicating strong fluorescence quenching.
**Figure S8 (A)** Example_1 and (**B)** Example_2 of a shifted absorption scan curve. The insets show a higher magnification. The white and black circles are the duplicates with ethanol and the white and black triangles with 1 mM oABA. One curve with oABA is clearly shifted over the entire absorption range. This is clearly not a specific signal and must be corrected.
**Figure S9** Correlation between the age of 44 child and adolescent control urine samples (6‐17 HV and < 6 HV cohorts) and the creatinine concentration in mg/dl. The higher μmol/mmol creatinine P6C concentrations in children below 3 years of age might be partially caused by the low creatinine concentrations.
**Table S1**: Comparison of different published AASA/P6C quantification methods
**Table S2:** Apparent extinction coefficient adjustment to allow proper comparison of data
**Table S3:** Entire absorption curve shifting artefact
**Table S4:** Influence of the creatinine levels on the final P6C concentrations using the examples from Figure S8 and Table S3
