# Supplementary material for: Condensation of delta‐1‐piperideine‐6‐carboxylate with ortho‐aminobenzaldehyde allows its simple, fast, and inexpensive quantification in the urine of patients with antiquitin deficiency
Source: J Inherit Metab Dis. 2020 Jan 29;43(4):891–900. doi: 10.1002/jimd.12214 (PMC7384183; doi:10.1002/jimd.12214)
Supplement: Supplementary file 1 — FIGURE S1 Absorption and fluorescence characterisation of the fusion product of P6C with oABA. (A) Absorption spectrum of 200 μM PC6 with and without preincubation with 1 mM oABA in 20 mM Hepes pH 7.0 for 2 hours at room temperature; (B) Excitation scan between 360 and 580 nm and fixed emission at 620 nm; (C) Excitation at 460 nm and emission scan between 500 and 700 nm; (D) Specific emission signal generated by subtracting the P6C/0.5% ethanol (oABA matrix) from the P6C/oABA RFUs; black squares (■) P6C incubated with oABA; white squares (□) P6C with ethanol (oABA matrix); black triangles (▲) H2O with oABA; white triangles (Δ) H2O with 0.5% ethanol; oABA, ortho‐aminobenzaldehyde; P6C, Delta‐1‐piperideine‐6‐carboxylate; RFU, Relative Fluorescence Units; ABS, Absorption Figure S2 Identification of the predicted P6C/oABA fusion product using ESI mass spectrometry. (A) P6C (m/z 128.1 Da) incubated with 0.5% ethanol (oABA matrix) in Hepes buffer; (B) P6C region enlarged; (C) P6C incubated with oABA with the expected new signal of the oABA/P6C condensate at 231.22 Da; the second new peak at 253.19 probably represents a sodium adduct (+22 Da); The main peaks at 239.16 and 260.87 Da are caused by Hepes and its sodium adduct; (D) MS/MS of the 231.22 Da peak with expected fragments of 106.1, 144.1 and 186.2 Da. (E) Relevant chemical structures including some MS2 fragments; oABA, ortho‐aminobenzaldehyde; P6C, Delta‐1‐piperideine‐6‐carboxylate; cps, counts per second Figure S3 P6C concentrations are not elevated in plasma of ATQ patients, but response rates of spiked P6C are high. (A) Example of a representative plasma standard curve after spiking different concentrations of P6C into EDTA plasma of a healthy volunteer (HV); the mean of duplicates is shown after subtracting fluorescence from the control sample with 0.5% ethanol (oABA matrix); R = 0.99; (B) Endogenous P6C concentrations in 7 HVs (black bars) and response after 20 μM P6C spiking (grey bars); Mean (+/‐SEM) response [file JIMD-43-891-s001.docx]

**SUPPORTING INFORMATION**

**Condensation of delta-1-piperideine-6-carboxylate with ortho-aminobenzaldehyde allows its simple, fast and inexpensive quantification in the urine of patients with Antiquitin deficiency**

Thomas Boehm^1^, Holger Hubmann^2^, Karin Petroczi^1^, Déborah Mathis^3^, Kristaps Klavins^4^, Guenter Fauler^5^, Barbara Plecko^2^, Eduard Struys^6^, Bernd Jilma^1^

^1^Department of Clinical Pharmacology, Medical University of Vienna, Waehringer Guertel 18-20, 1090 Vienna, Austria

^2^Department of Pediatrics and Adolescent Medicine, Division of General Pediatrics, Medical University of Graz, 8036 Graz, Austria

^3^Department of Clinical Chemistry and Biochemistry, University Children's Hospital Zurich, 8032 Zurich, Switzerland

^4^CeMM Research Centre for Molecular Medicine of the Austrian Academy of Sciences, Lazarettgasse 14, 1090 Vienna, Austria

^5^Clinical Institute of Medical and Chemical Laboratory Diagnostics, Medical University of Graz, 8036 Graz, Austria

^6^Department of Clinical Chemistry, Amsterdam University Medical Centers, location VUmc, Amsterdam, The Netherlands

**Correspondence**

Thomas Boehm

Department of Clinical Pharmacology, Medical University of Vienna

Waehringer Guertel 18-20, 1090 Vienna, Austria

Tel.: +43-1-40400-49580, Fax: +43-1-40400-29980

Email: [thomas.boehm@meduniwien.ac.at](mailto:thomas.boehm@meduniwien.ac.at)

**1 | SUPPLEMENTARY METHODS**

**1.1 | Identification of CHHPQ by electrospray ionization mass spectrometry (ESI-MS)**

The triple aromatic ring structure of the condensate between oABA and delta-1-pyrroline was proposed in 1936, but never analyzed using NMR or mass spectrometry (Schoepf and Oechler 1936). The IUPAC name for the fusion of oABA with P6C is 9‑carboxy-5,5a,6,7,8,9-hexahydropyrido[2,1-b]quinazoline-10-ium and CHHPQ is used as abbreviation (see Figure S2 for the structure). A final concentration of 150 µM P6C was incubated in 20 mM Hepes buffer (H3375, Sigma-Aldrich, Vienna, Austria) pH 7.0 with and without a final concentration of 1 mM oABA (A9628, Sigma-Aldrich, Vienna, Austria) for 2 hours at room temperature. The absorption maximum of oABA is at 360 nm. Successful fusion was verified by absorbance and fluorescence measurements (data not shown). The samples were analyzed using direct infusion mass spectrometry (MS). A Xevo TQ-MS mass spectrometer equipped with electrospray ionization source (ESI) was employed for the analysis. A Hamilton syringe pump was used to introduce samples into the mass spectrometer at a flow rate of 10 µl per minute. The mass spectrometer settings were as follows: positive ionization mode; source temperature: 150 °C; capillary voltages: 3.5 kV; desolvation temperature: 200 °C; cone voltage: 20 V. The mass spectra were acquired between 80 and 400 m/z with a scan speed of 1 second and an acquisition time of 30 seconds. Collision‑induced dissociation using argon as the collision gas and a collision energy of 30 arbitrary units was used to obtain MS2 spectra. Raw data files were used for preparing the figures in Excel. Chemicals were drawn with MarvinSketch.

**1.2 | Inhibition of the oABA/P6C fusion reaction by the Knoevenagel condensate (PLP/P6C)**

To test the influence of the formation of a Knoevenagel condensate on the fusion propensity of oABA with P6C a final concentration of 40 µM P6C was incubated in 20 mM Hepes buffer pH 7.0 with water or freshly prepared 80 or 240 µM PLP (P3657, Sigma-Aldrich, Vienna, Austria). The absorption maximum of PLP is at 388 nm. As controls we used reactions without P6C and without PLP. All samples were incubated in the dark at 37°C for 24 hours. After a short spin to collect the fluid droplets from the top of the Eppendorfs, 10 µl of a 20 mM oABA solution was added to all samples except the control sample which consisted of just Hepes buffer and water. These mixtures were incubated for 1 hour at ambient temperature before absorption and fluorescence readings were performed as described below at pH 7.0 and additionally at pH 0.7 (200 mM HCl). For the reverse experiment a final concentration of 20 µM P6C was first incubated for 90 minutes at room temperature (RT) with 1 mM oABA, followed by incubation at 37°C in the dark for 24 hours after adding PLP at a final concentration of 200 µM. Absorption and fluorescence readings were performed as described below at pH 7.0 and also pH 0.7.

**1.3 | Analysis of endogenous and exogenous (spiked) P6C in EDTA or Heparin plasma**

EDTA plasma samples were obtained from healthy volunteers to measure baseline values of P6C. Five control EDTA plasma samples were also obtained from the Children’s Hospital in Graz. A total of 9 plasma samples from 7 ATQ patients collected at two hospitals (Graz, Austria and Zurich, Switzerland) were included. Relevant patient characteristics are described in Table 1 of the main manuscript. Ninety µl EDTA or heparin (samples from Zurich) plasma were mixed with 5 µl 10% ethanol or 20 mM oABA and 5 µl water or exogenous P6C. All samples were analyzed in duplicate. There was no measurable difference between EDTA and heparin plasma (data not shown). After incubation for 1 hour at RT in the dark, 200 µl 7.5% TCA (99.5% trichloroacetic acid, 91228, Sigma-Aldrich, Vienna, Austria) was added and incubated for 20 minutes on ice. After high speed centrifugation for 10 minutes, 200 µl were recovered and the pH adjusted to 4.0 with 5.2 µl 10 M NaOH and 50 µl 500 mM citrate buffer pH 4.0. To reduce the pH to 0.7, a 2 M HCl solution was added to obtain a final HCl concentration of 200 mM. TCA precipitation is necessary to reduce protein autofluorescence interference. Relative fluorescence units (RFUs) were measured with a Synergy^TM^ H1 Multi-Mode Microplate reader using a custom filter cube and monochromator‑based excitation and emission scans. The custom filter cube is composed of an excitation filter 440/30 nm (range 425 to 455 nm), a dichroic mirror with a cut-off of 550 nm and an emission filter of 620/40 nm (range 600 to 640 nm). This filter cube was selected based on the absorption maximum of the condensation product of delta-1-pyrroline with oABA at 440 nm. A filter with an excitation maximum at 460 nm (range 440 to 480 nm) might increase the sensitivity to measure CHHPQ, because the area under the curve (AUC) for absorption by the delta-1-piperideine/oABA fusion product is increased by about 50%, whereas the control AUCs or putrescine AUCs are unchanged switching from the 440 to 460 nm filter set (data not shown). We tested such a filter and the fluorescence was increased by 43% (data not shown). Emission scans were performed using 4 nm steps with excitation at 460 nm and emission scan from 500 to 700 nm. For excitation scans we used fixed emission at 620 nm after excitation from 350 to 580 nm. For fluorescent measurements 100 µl of the reaction mixture used for absorption measurements (see main manuscript) were diluted with 100 µl water and the 200 µl were measured in black fluorescent plates (Corning-Costar® 96-well Black Flat Bottom Polystyrene microplates 3915, Szabo-Scandic, Vienna, Austria) using a custom filter cube (see above) or the monochromator from the Synergy^TM^ H1 microplate reader. For low pH measurements 2 M HCl was added to obtain a final concentration of 200 mM HCl.

**2 | SUPPLEMENTARY RESULTS**

**2.1 | Absorption and fluorescence properties of the fusion product CHHPQ (oABA/P6C)**


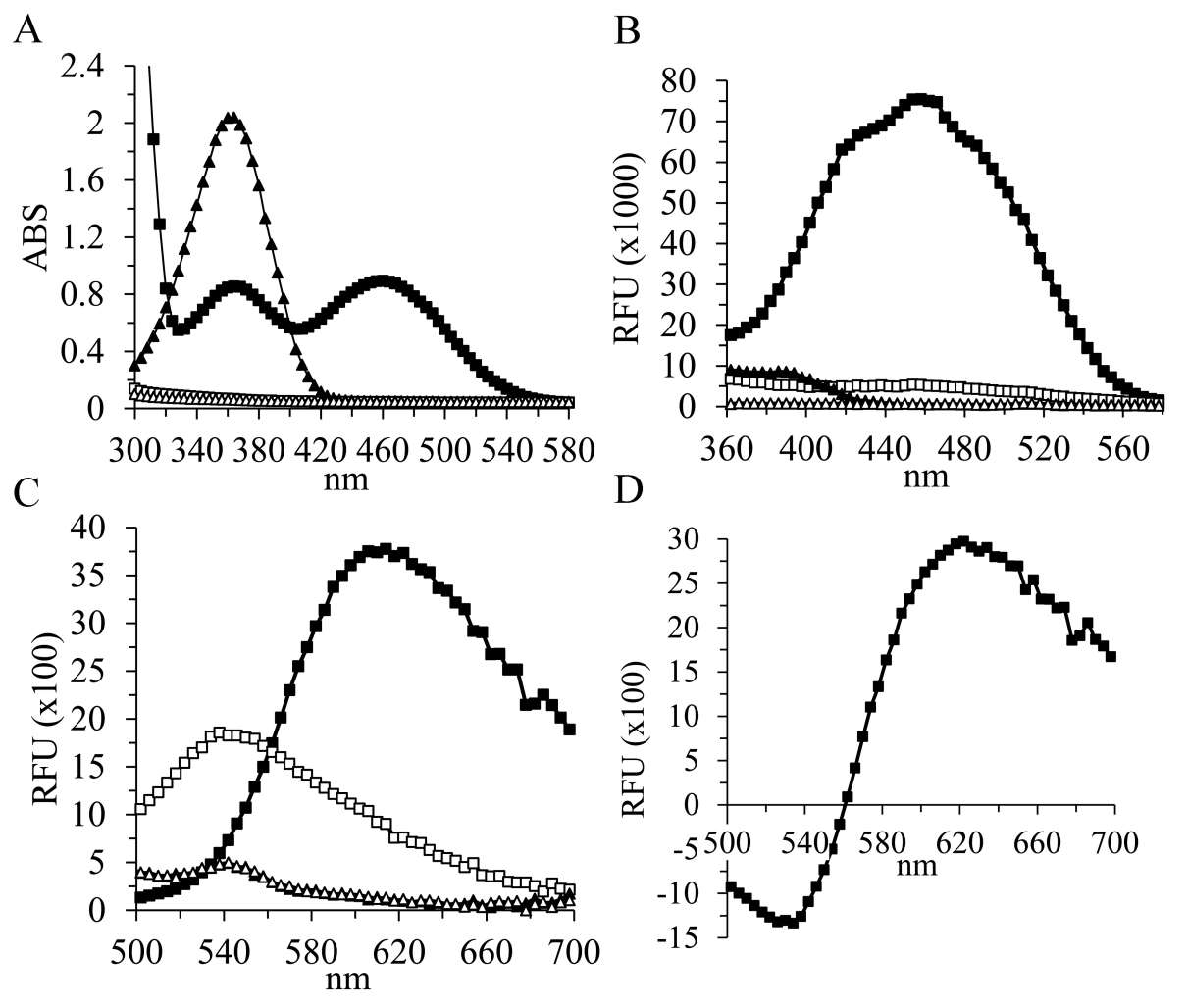


**FIGURE S1** Absorption and fluorescence characterization of the fusion product of P6C with oABA. **(A)** Absorption spectrum of 200 µM PC6 with and without preincubation with 1 mM oABA in 20 mM Hepes pH 7.0 for 2 hours at room temperature; **(B)** Excitation scan between 360 and 580 nm and fixed emission at 620 nm; **(C)** Excitation at 460 nm and emission scan between 500 and 700 nm; **(D)** Specific emission signal generated by subtracting the P6C/0.5% ethanol (oABA matrix) from the P6C/oABA RFUs; black squares (■) P6C incubated with oABA; white squares (□) P6C with ethanol (oABA matrix); black triangles (▲) H_2_O with oABA; white triangles (Δ) H_2_O with 0.5% ethanol; oABA, ortho-aminobenzaldehyde; P6C, Delta-1-piperideine-6-carboxylate; RFU, Relative Fluorescence Units; ABS, Absorption

**2.2 | Identification of the oABA/P6C condensate using ESI-MS**

**
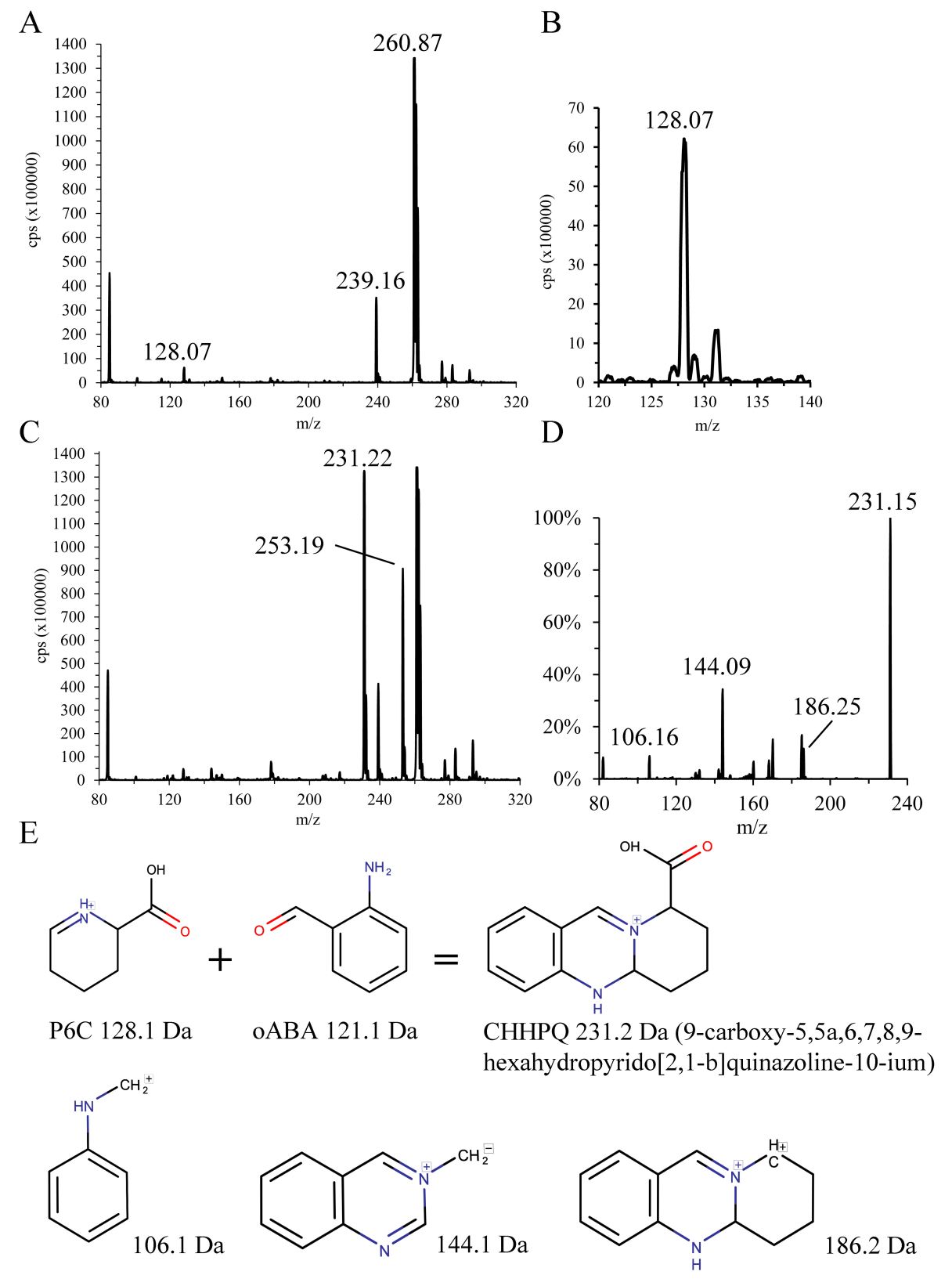
**

**FIGURE S2** Identification of the predicted P6C/oABA fusion product using ESI mass spectrometry. **(A)** P6C (m/z 128.1 Da) incubated with 0.5% ethanol (oABA matrix) in Hepes buffer; **(B)** P6C region enlarged; **(C)** P6C incubated with oABA with the expected new signal of the oABA/P6C condensate at 231.22 Da; the second new peak at 253.19 probably represents a sodium adduct (+22 Da); The main peaks at 239.16 and 260.87 Da are caused by Hepes and its sodium adduct; **(D)** MS/MS of the 231.22 Da peak with expected fragments of 106.1, 144.1 and 186.2 Da. **(E)** Relevant chemical structures including some MS2 fragments; oABA, ortho-aminobenzaldehyde; P6C, Delta-1-piperideine-6-carboxylate; cps, counts per second

**2.3 | P6C concentrations in plasma of healthy volunteers and ATQ-deficient subjects**

EDTA plasma samples from healthy volunteers were used to measure baseline values of P6C. Five control EDTA plasma samples were also obtained from the Children’s Hospital in Graz. A total of 9 plasma samples from 7 ATQ patients collected at two hospitals (Graz, Austria and Zurich, Switzerland) were included. Relevant patient characteristics are described in Table 1 of the main manuscript.

Emission and excitation scans of the condensate between oABA and P6C are very similar to the fusion product of oABA and delta-1-piperideine (data not shown). Delta-1-piperideine can be readily generated by incubating cadaverine with recombinant human diamine oxidase (DAO). Incubation of human plasma with 200 µM cadaverine or putrescine without exogenous DAO does not lead to any increase in fluorescence during 3 hours of incubation at 37°C, indicating that the generation of a fluorescent fusion product of P6C with oABA is very specific (data not shown).

Based on published P6C plasma concentrations in ATQ-deficient patients (Sadilkova et al 2009; Yuzyuk et al 2016a) with a minimum/maximum range of 3 to 57 µM, we expected elevated P6C concentrations in the 9 samples from the 7 ATQ patients. A representative standard curve after spiking different P6C concentrations into EDTA plasma of a healthy volunteer is shown in Figure S3A. The negative control consisting of 10% ethanol instead of 1 mM oABA dissolved in 10% ethanol was subtracted from the samples with oABA and P6C. In Figure S3B the endogenous P6C concentrations of 7 healthy volunteers were determined. The mean (+/-SEM) response after spiking 20 µM P6C into the same plasma samples was 80% (9%; Figure S3B). The endogenous P6C concentrations of 9 samples from 7 different ATQ subjects are presented in Figure S3C. Although there is a trend towards higher concentrations with a p-value of 0.083 comparing the ATQ P6C concentrations with the HV and Graz control samples G_C, only one ATQ subject demonstrated clearly elevated P6C concentrations (Figure S3C). High response rates after spiking of exogenous P6C into plasma of ATQ patients were measured in 4 ATQ patient samples (Figure S3D). For the other 3 ATQ cases we did not have enough plasma for testing.





**FIGURE S3** P6C concentrations are not elevated in plasma of ATQ patients, but response rates of spiked P6C are high. **(A)** Example of a representative plasma standard curve after spiking different concentrations of P6C into EDTA plasma of a healthy volunteer (HV); the mean of duplicates is shown after subtracting fluorescence from the control sample with 0.5% ethanol (oABA matrix); R=0.99; **(B)** Endogenous P6C concentrations in 7 HVs (black bars) and response after 20 µM P6C spiking (grey bars); Mean (+/-SEM) response was 80% (9%); **(C)** P6C plasma concentrations of 9 samples from 7 different ATQ subjects (Zurich samples have been measured only once and Z1_2 only in singlicate; the mean (+/-SEM) of the duplicates are shown; Graz samples have been measured once in duplicate and the mean (+/-SEM) are shown) and healthy controls (HV; n=7; different HV samples compared to (**B**) were used; G_C; n=5; Graz control plasma samples from five subjects <18 years); the means of duplicates (+/-SEM) are shown; **(D)** Response of spiked P6C in 4 ATQ subjects normalized to 3 HVs in one experiment and 2 HVs samples in a second experiment; we did not have enough plasma from the Zurich samples to perform further experiments; the means of duplicates (+/-SEM) are shown; oABA, ortho-aminobenzaldehyde; P6C, Delta-1-piperideine-6-carboxylate; RFU, Relative Fluorescence Units

**2.3 | Interference of the Knoevenagel condensate with oABA fusion**

We hypothesized that in plasma P6C is completely fused with PLP and therefore cannot condensate with oABA anymore. To test this hypothesis we performed two types of experiments. First, a 2- and 6-fold molar excess of PLP over P6C was incubated overnight at 37°C and afterwards 1 mM oABA was added for 90 minutes at ambient temperature. The data indicate that preincubation of P6C with PLP strongly inhibited condensation of oABA with the P6C/PLP Knoevenagel fusion product (Figure S4). We measured absorption and fluorescence at pH 7.2 and 0.7 (200 mM HCl) because the strong absorption of PLP and oABA in the 360 to 390 nm range shows some interference at pH 7.2, which appears to be largely eliminated at pH 0.7. This experiment was repeated with a 10-fold molar excess of PLP with similar results (Figure S5). In the second type of experiment we first incubated oABA with P6C to form the triple aromatic structure CHHPQ and afterwards added a 10-fold molar excess of PLP. Under these circumstances the absorption and fluorescence signals are inhibited by 80%, indicating that PLP still seems able to fuse to the oABA/P6C condensation product (Figure S4E; Figure S6). The absorption and fluorescence percent inhibition data are summarized in Figure S4E. The Knoevenagel condensate of P6C with PLP does not seem to allow fusion with oABA and therefore does not generate the characteristic absorption and fluorescence signal. This might explain why only 1 plasma sample from 7 ATQ patients showed elevated P6C concentrations. In this case excess free P6C was possibly still available to condensate with oABA.


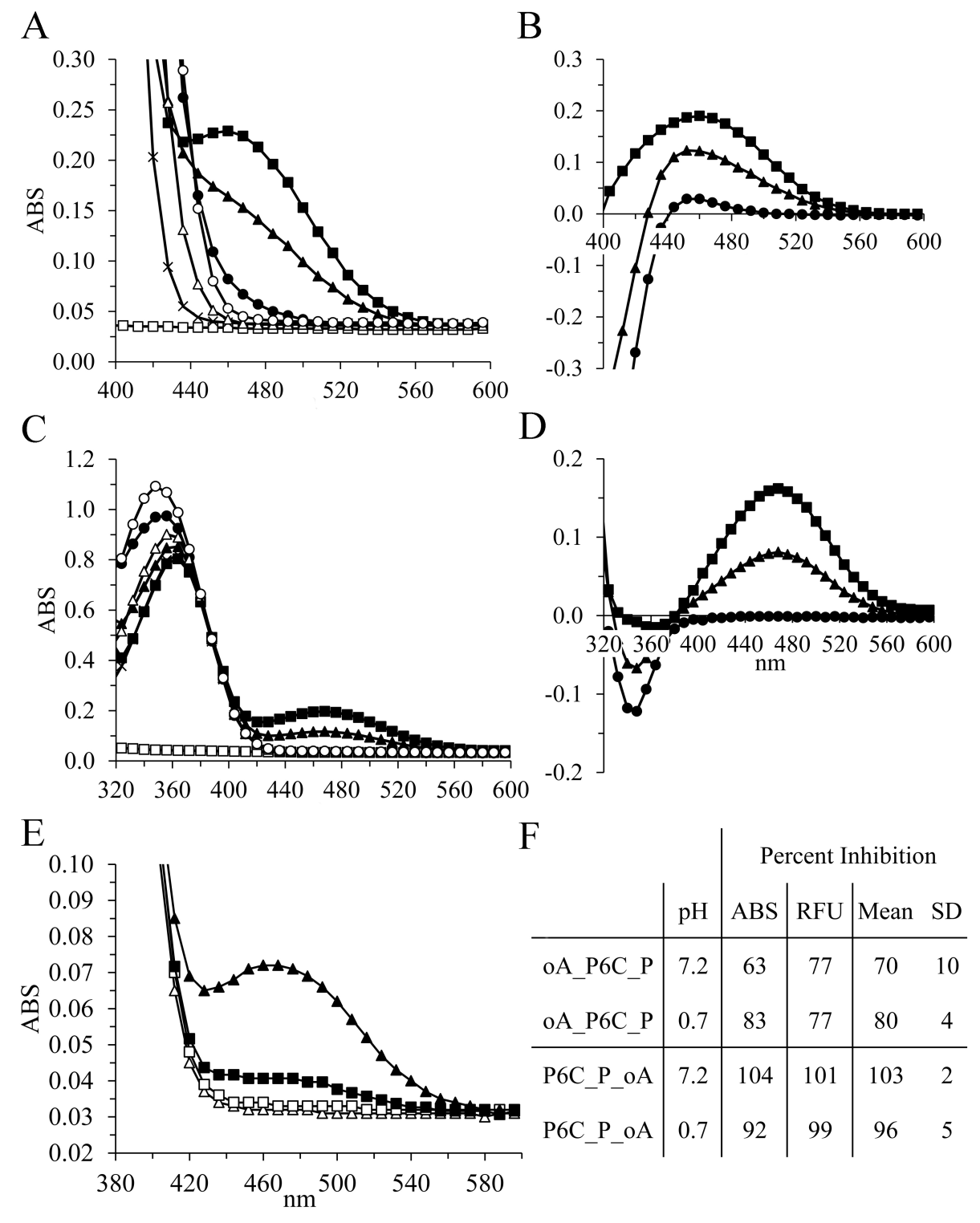


**FIGURE S4** P6C Knoevenagel condensation with PLP does not allow subsequent oABA fusion. **(A)** 40 µM P6C was incubated with a 2- and 6-fold excess of PLP overnight at 37°C followed by incubation with oABA for 90 minutes. Absorption was measured at pH 7.2; **(B)** Specific signal of **(A)** after subtracting the relevant controls; **(C)** Absorption measurements at pH 0.7; the low pH reduced the PLP and oABA signal about 5-fold with a minimal effect on the P6C signal; **(D)** Specific signal of **(C)** after subtracting the relevant controls; In **(A)** to **(D)** white squares (□) represent P6C incubated with 0.5% ethanol (oABA matrix); black squares (■) P6C with oABA; black triangles (▲) P6C with a 2‑fold molar excess of PLP followed by oABA; black circles (●) P6C with a 6-fold molar excess of PLP followed by oABA; crosses (x) just water with 0.5% ethanol; white triangles (Δ) and white circles (○) 80 and 240 µM PLP followed by oABA; **(E)** P6C was first incubated with oABA for 90 minutes at room temperature and afterwards a 10-fold excess of PLP was added for 24 hours at 37°C before absorption measurements at room temperature and pH 0.7; white triangles (Δ) represent oABA in Hepes buffer; white squares (□) oABA incubated with PLP; black triangles (▲) oABA with P6C and black squares (■) oABA with P6C followed by a 10-fold excess of PLP; **(F)** Summary of percent inhibition using absorption and fluorescence measurements at pH 7.2 and pH 0.7 incubating P6C first with oABA followed by PLP (oA_P6C_P) or P6C first with PLP followed by oABA after 24 hours (PC6_P_oA); Percentage inhibition was calculated using absorption data at 460 nm and fluorescence data with the custom filter cube after subtracting the appropriate controls; oABA, ortho-aminobenzaldehyde; P6C, Delta-1-piperideine-6-carboxylate; PLP, Pyridoxal 5-Phosphate; RFU, Relative Fluorescence Units; SD, Standard deviation; ABS, Absorption


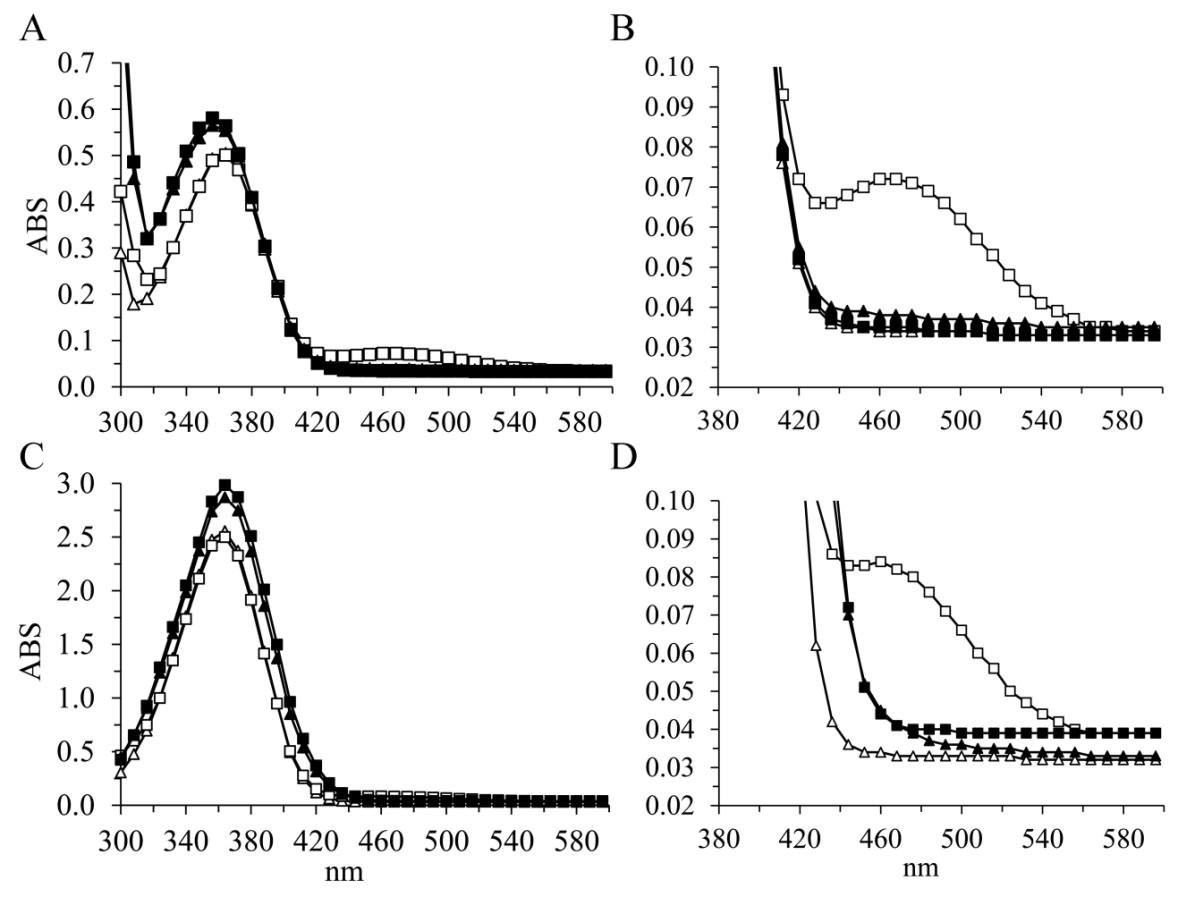


**FIGURE S5** P6C Knoevenagel condensation with PLP does not allow subsequent oABA fusion - Repeat. **(A)** 20 µM P6C was incubated with a 10-fold excess of PLP for 24 hours at 37°C; 1 mM oABA was added for 90 minutes and absorption measured at pH 0.7; **(B)** Magnification of the 460 nm region of **(A)**; **(C)** same as **(A)** but measured at pH 7.2; **(D)** Magnification of the 460 nm region; white triangles (Δ) represent oABA with Hepes buffer (20 mM; pH 7.0); white squares (□) P6C incubation with oABA; black triangles (▲) P6C with a 10-fold excess of PLP for 24 hours followed by oABA; black squares (■) PLP with oABA; oABA, ortho-aminobenzaldehyde; P6C, Delta-1-piperideine-6-carboxylate; PLP, Pyridoxal 5’-phosphate; ABS, Absorption


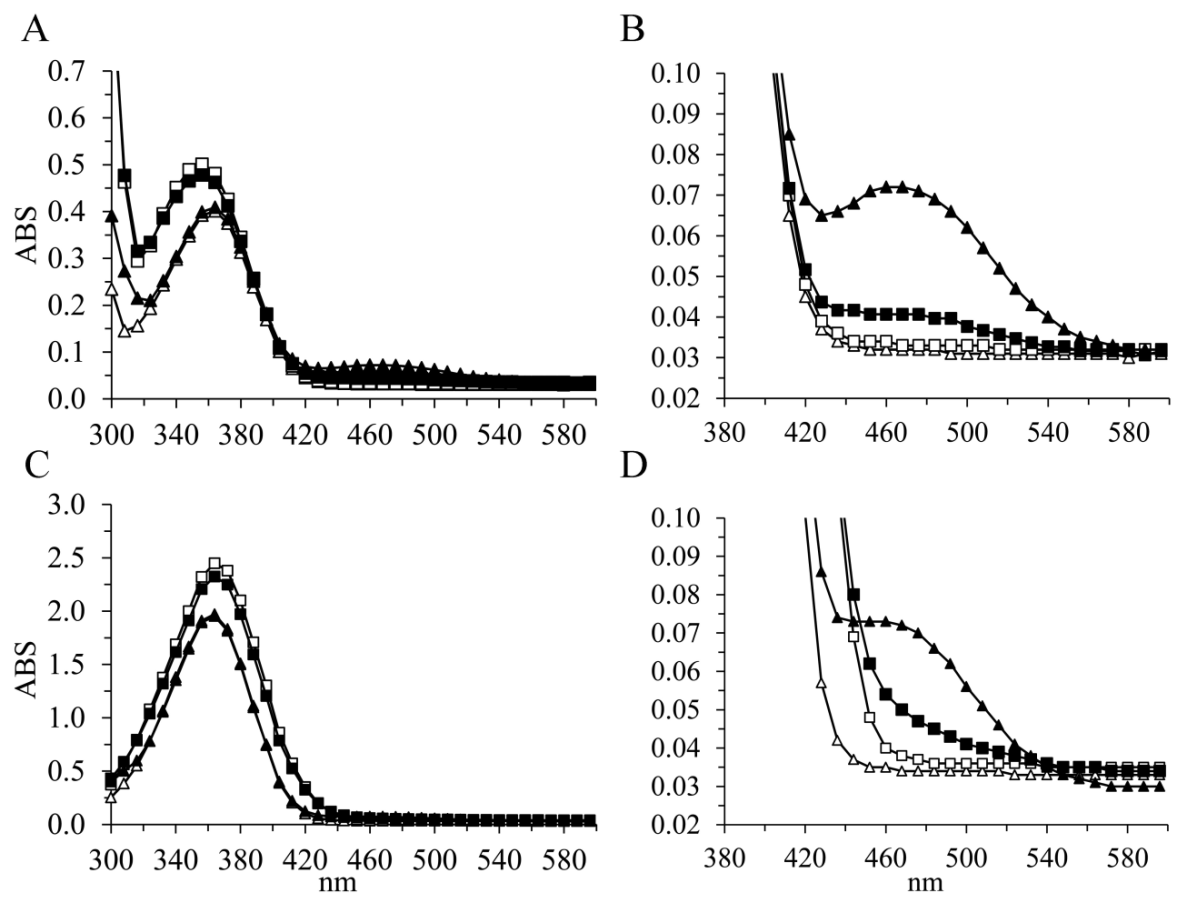


**FIGURE S6** PLP seems to be able to form a Knoevenagel condensate with the P6C/oABA fusion product and inhibits absorption and fluorescence. **(A)** 20 µM P6C was first incubated with oABA for 90 minutes at room temperature and afterwards a 10-fold excess of PLP was added for 24 hours at 37°C and absorption measured at pH 0.7; **(B)** Magnification of the 460 nm region of **(A)** (also shown in Figure S4E but repeated here for consistency); **(C)** same as **(A)** but measured at pH 7.2; **(D)** Magnification of the 460 nm region of **(C)**; white triangles (Δ) represent incubation of oABA with Hepes buffer (20 mM; pH 7.0); white squares (□) oABA with PLP; black triangles (▲) oABA with P6C and black squares (■) oABA with P6C followed by a 10-fold excess of PLP; oABA, ortho-aminobenzaldehyde; P6C, Delta-1-piperideine-6-carboxylate; PLP, Pyridoxal 5’-phosphate; ABS, Absorption

**2.4 | Not all ATQ urine samples are amenable for fluorescence measurements**

Fluorescence measurements in minimally diluted urine are not straightforward. Some urine samples consistently produce negative fluorescence units after addition of oABA indicating fluorescence quenching (data not shown). Higher urine dilutions reduce quenching but also reduce specific signal generation. We obtained the best results with a urine dilution of at least 4‑fold and at a pH of 0.7. Under these conditions the standard curves spiking P6C into urine samples from healthy volunteers are reproducible (Figure S7A). There is also a high correlation between calculated µM P6C concentrations measured using absorption and fluorescence after removing two ATQ samples, which consistently showed negative fluorescence (Figure S7B). Nevertheless, P6C concentrations using absorption measurements are almost 1.5-fold higher indicating still relevant quenching of urine components. A liquid chromatography column coupled with fluorescence detection might significantly improve fluorescence based quantification of P6C in urine.

**
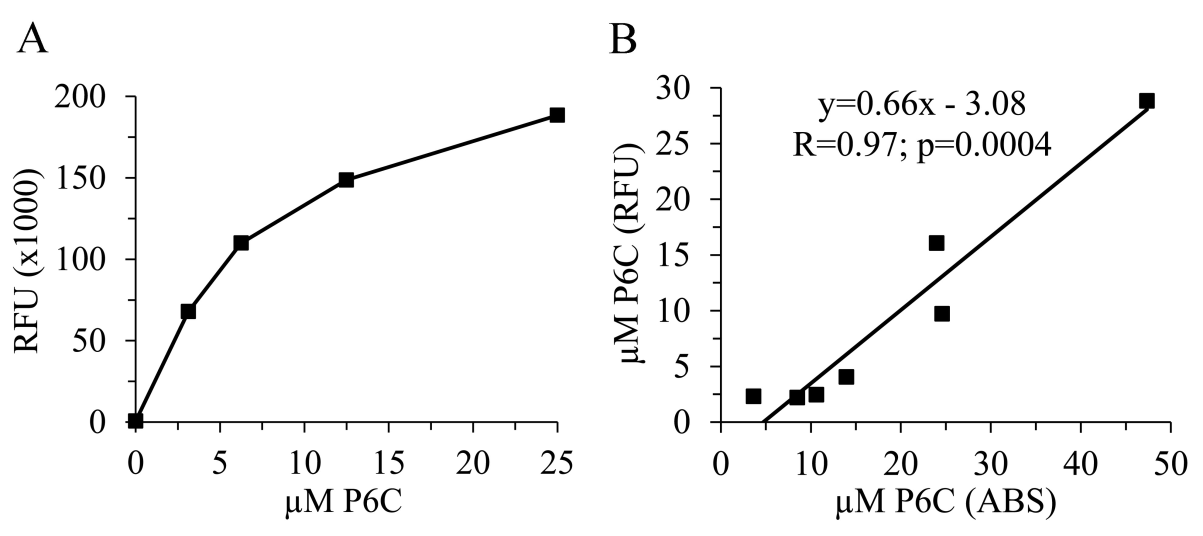
**

**FIGURE S7** Fluorescence based measurements of the oABA/P6C condensate are not straightforward in urine samples from ATQ patients. **(A)** The fluorescence standard curve after spiking different concentrations of exogenous P6C into the urine of a healthy volunteer; the control sample without addition of P6C was subtracted; 4PL non-linear regression analysis (R=0.99) was used to calculate the µM P6C concentrations of the ATQ subjects shown in (**B**); (**B)** Regression analysis between absorption and fluorescence µM concentrations excluding G205 and Z2_2 ATQ subject samples. Both show a negative signal after subtracting the ethanol autofluorescence signal from the signal in the presence of oABA indicating strong fluorescence quenching.

**Table S1**: Comparison of different published AASA/P6C quantification methods

| Method | Matrix | AASA and/or P6C measured | Normal AASA/P6C* levels | Mean*; Min/Max or Range ATQ | Number of ATQ patients | Reference |
| --- | --- | --- | --- | --- | --- | --- |
| oABA | urine | P6C | <1.33^$^ | 6; 2.9/12.3 | 9 (7)*** | This study |
| LC-MS/MS | urine | AASA/P6C | <2.2^§^ | nd; 44/388 | 7 | Yuzyuk 2016b |
| LC-MS/MS | urine | AASA | <1.5^$§^ | 23; 9.1/34 | 3 | Ferrer-Lopez 2014 |
| LC-MS/MS | urine | AASA | <3.3^$§§§^ | 106; 9/246 | 8 | Ferrer-Lopez 2014 |
| LC-MS/MS | urine | AASA | <0.5^§^ | nd; 0.6/5.8 | 40 | Struys 2012 (MM) |
| LC-MS/MS | urine | P6C | <0.05^§^ | nd; 0.2/8.6 | 40 | Struys 2012 |
| LC-MS/MS | urine | AASA | <1 | 23; 4/75 | 10 | Bok 2007 (MM) |
| LC-MS/MS | urine | AASA | <1 | 18; 1.6/342** | 12 | Plecko 2007 (MM) |
| LC-MS/MS | urine | AASA | <1 | 12; 7.5/168** | 11 | Mills 2006 |
| oABA | plasma | P6C | <5.2^$^ | 4.3; 2.0/11.5 | 9 (7)*** | This study |
| LC-MS/MS | plasma | AASA/P6C | <2.6^§^ | nd; 21/57 | 13 | Yuzyuk 2016b |
| LC-MS/MS | plasma | AASA | <0.2^§§^ | 4; 0.9/7 | 5 | Sadilkova 2009 |
| LC-MS/MS | plasma | P6C | <1.7^§§^ | 13; 3/28.4 | 5 | Sadilkova 2009 |
| LC-MS/MS | plasma | AASA | <0.2 | 4; 0.8/8 | 10 | Bok 2007 (MM) |
| LC-MS/MS | plasma | AASA | <0.2 | 4; 0.5/14 | 16 | Plecko 2007 (MM) |
| LC-MS/MS | plasma | AASA | <0.2 | 3.5; 1.5/4.6 | 6 | Mills 2006 |

*µmol/mmol creatinine in urine and µM in plasma; **Median instead of mean because of extreme outliers; ***Nine samples from 7 different ATQ patients; ^§^Normal values for subjects >1 year old; ^§§^Normal values for subjects >1 week; ^§§§^Normal values for subjects 0 to 0.5 years; ^$^Prediction interval 99% using all control values (n=74); nd=not described; MM=Mills et al 2006 Method; In ^§^, ^§§^ and ^§§§^ it is not described whether the cut-off is the 99% confidence interval.

**2.5 | Stability of the P6C standard and therefore of the slope of the standard curve**

The following table (Table S2) shows the different apparent extinction coefficients of the standard curves using 0, 3.1, 6.3, 12.5 and 25 µM P6C in the same urine from a healthy volunteer frozen in single use aliquots in March 2019 using the final protocol in April/May 2019 and October 2019. Storing the P6C standard for an additional 6 months apparently caused the extinction coefficient to degrade significantly (p=0.013). To properly compare the April/May with the October data we adjusted the October data using the mean factor of 1.61. As can be seen in the table, the endogenous P6C concentrations increased because of the lower extinction coefficient. We repeated measurements in the Zurich samples again in October and they were also increased accordingly (data not shown). The relative difference between healthy volunteer samples and antiquitin patient samples is independent of the extinction coefficient. It might even be possible to perform this assay without P6C standard, if oABA testing can be performed using a different method.

It is critical that the P6C standard is stored at sufficiently low temperatures (below -70°C) and freshly prepared at appropriate intervals. A suitable commercial source of high quality P6C would be highly advantageous for clinical laboratories with limited chemistry expertise.

**Table S2:** Apparent extinction coefficient adjustment to allow proper comparison of data

| Date of experiment | Extinction coefficient* | endP6C µM | endP6C µmol/ mmol crea | R^2**^ |
| --- | --- | --- | --- | --- |
| 10Apr2019 | 4565 | 1.66 | 0.25 | 0.995 |
| 01May2019 | 4493 | 2.10 | 0.32 | 0.994 |
| 03May2019 | 3965 | 2.39 | 0.38 | 0.998 |
| 21May2019 | 3117 | 2.66 | 0.41 | 0.998 |
| Mean | 4035 | 2.20 | 0.34 |  |
|  |  |  |  |  |
| 23Oct2019 | 2545 | 4.09 | 0.65 | 0.999 |
| 24Oct2019 | 2706 | 3.50 | 0.56 | 0.999 |
| 28Oct2019 | 2301 | 3.29 | 0.50 | 0.997 |
| Mean | 2517 | 3.62 | 0.57 |  |
|  |  |  |  |  |
| 23Oct2019 |  |  | 1.59 |  |
| 24Oct2019 |  |  | 1.49 |  |
| 28Oct2019 |  |  | 1.75 |  |
| Average adjustment factor = | |  | 1.610 |  |

*The measured extinction coefficients have been adjusted to the 200 µl volume using half-area plates by multiplication with 0.85. The light path of UV half-area plates is 1 cm at 170 µl; **The squared correlation coefficients of the standard curves.

**2.6 | Shifting of the entire absorption scan curves**

A shift of the entire absorption curve by a few nm was observed in less than 5% of wells using UV half-area 96-well microtiter plates. In many cases this has no relevant effect on the results, but if the creatinine concentration is low, the P6C µmol/mmol creatinine concentration can easily double or triple. In Figure S8 two examples are shown. Tables S3 and S4 demonstrate the influence on measured P6C concentrations dependent on the creatinine levels.

This may be due to a batch problem with our UV plates, but for automated analysis similar observations with other microtiter plates must be properly corrected.

**

**

**FIGURE S8 (A)** Example_1 and (**B)** Example_2 of a shifted absorption scan curve. The insets show a higher magnification. The white and black circles are the duplicates with ethanol and the white and black triangles with 1 mM oABA. One curve with oABA is clearly shifted over the entire absorption range. This is clearly not a specific signal and must be corrected.

**Table S3:** Entire absorption curve shifting artifact

| Raw absorption data at 460 nm | | |  |  |
| --- | --- | --- | --- | --- |
|  | Ethanol | Ethanol | oABA | oABA |
| Example_1 | 0.039 | 0.040 | 0.041 | 0.048 |
| Example_2 | 0.132 | 0.133 | 0.136 | 0.151 |
|  |  |  |  |  |
|  | Mean Ethanol | Mean oABA unadjusted | Mean oABA adjusted |  |
| ABS_Ex_1 | 0.0395 | 0.0445 | 0.0410 | ABS |
| ABS_Ex_2 | 0.1325 | 0.1435 | 0.1360 | ABS |
|  | Delta | 0.005 | 0.0015 | ABS |
|  | Delta | 0.011 | 0.0035 | ABS |
| P6C_Ex_1* |  | 1.25 | 0.38 | µM |
| P6C_Ex_2* |  | 2.75 | 0.88 | µM |

*4000 M^-1^cm^-1^ used as extinction coefficient

**Table S4:** Influence of the creatinine levels on the final P6C concentrations using the examples from Figure S8 and Table S3

|  |  | µmol P6C/mmol crea | | µmol P6C/mmol crea | |
| --- | --- | --- | --- | --- | --- |
| Creatinine |  | Ex_1 | Ex_2 | Ex_1 | Ex_2 |
| mg/dl | mmol/l | unadjusted | unadjusted | adjusted | adjusted |
| 100 | 8.8 | 0.14 | 0.31 | 0.04 | 0.10 |
| 80 | 7.1 | 0.18 | 0.39 | 0.05 | 0.12 |
| 40 | 3.5 | 0.35 | 0.78 | 0.11 | 0.25 |
| 20 | 1.8 | 0.71 | 1.56 | 0.21 | 0.49 |
| 15 | 1.3 | 0.94 | 2.07 | 0.28 | 0.66 |
| 10 | 0.9 | 1.41 | 3.11 | 0.42 | 0.99 |

**
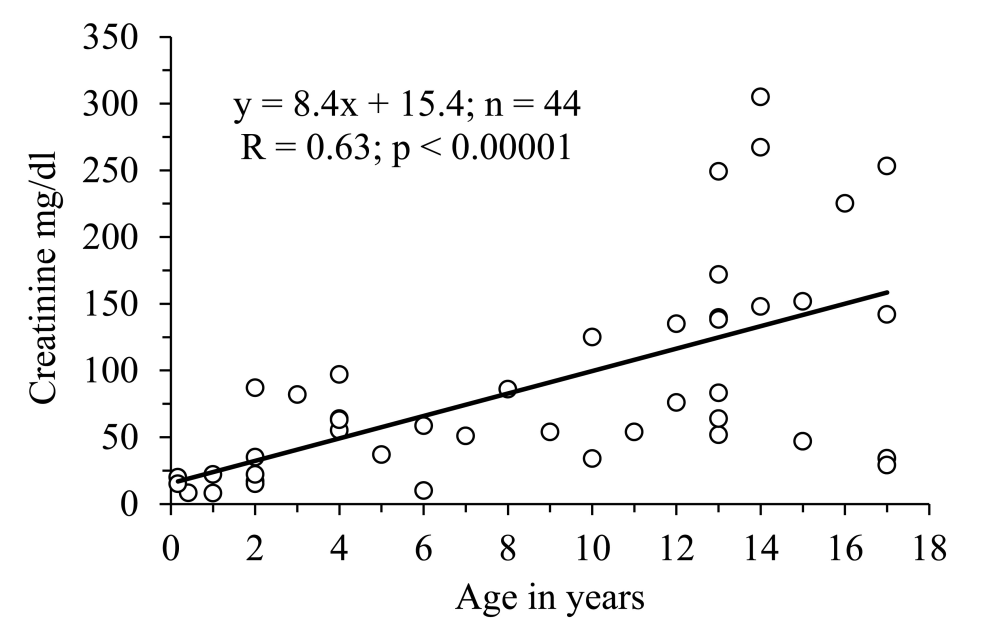
**

**FIGURE S9** Correlation between the age of 44 child and adolescent control urine samples (6-17HV and <6HV cohorts) and the creatinine concentration in mg/dl. The higher µmol/mmol creatinine P6C concentrations in children below 3 years of age might be partially caused by the low creatinine concentrations.
